# Luteinizing hormone and androstendione are independent predictors of ovulation after laparoscopic ovarian drilling: a retrospective cohort study

**DOI:** 10.1186/1477-7827-7-153

**Published:** 2009-12-30

**Authors:** Johannes Ott, Stefan Wirth, Kazem Nouri, Christine Kurz, Klaus Mayerhofer, Johannes C Huber, Clemens B Tempfer

**Affiliations:** 1 Department of Gynecologic Endocrinology and Reproductive Medicine, Medical University of Vienna, Vienna, Austria

## Abstract

**Background:**

Our objective was to investigate luteinizing hormone, follicle-stimulating hormone, testosterone, and androstenedione as predicitve markers for ovulation after laparoscopic ovarian drilling.

**Methods:**

We retrospectively analyzed 100 clompihen-resistant patients with the polycystic ovary syndrome who underwent laparoscopic ovarian drilling at our department. The main outcome measure was spontaneous postoperative ovulation within three months after laparoscopic ovarian drilling. In order to predict spontaneous ovulation, we tested the following parameters by use of a univariate followed by a multivariate regression model: Preoperative serum levels of LH, FSH, testosterone, and androstenedione as well as patients' age and body mass index. In addition, we focused on pregnancy and life birth rates.

**Results:**

Spontaneous ovulation was documented in 71/100 patients (71.0%). In a univariate and multivariate analysis, luteinizing hormone (OR 1.58, 95%CI: 1.30-1.92) and androstenedione (OR 3.03, 95%CI: 1.20-7.67), but not follicle-stimulating hormone and testosterone were independent predictors of ovulation. Using a cut-off for luteinizing hormone and androstenedione of 12.1 IU/l and 3.26 ng/ml, respectively, spontaneous ovulation was observed in 63/70 (90.0%) and 36/42 patients (85.7%) with elevated and in 8/30 (26.7%) and 35/58 (60.3%) patients with low luteinizing hormone and androstenedione levels, respectively. The sensitivity, specificity, positive and negatvie predictive values for luteinizing hormone and androstendione as predictors of spontaneous ovulation after ovarian drilling were 88.7% (95%CI: 79.0-95.0%), 75.9% (95%CI: 56.5-89.7%), 90.0% (95%CI: 80.5-95.8%), and 73.3% (95%CI: 54.1-87.7%) for luteinizing hormone, and 50.7% (95%CI: 38.6-62.8%), 79.3% (95%CI: 60.3-92.0%), 85.7% (95%CI: 71.5-94.6%), and 39.7% (95%CI: 27.0-53.4%) for androstenedione, respectively. Complete one-year follow-up was available for 74/100 patients (74%). We observed a one-year pregnancy rate and a resulting life-birth rate of 61% and 51%, respectively.

**Conclusions:**

Luteinizing hormone and androstenedione prior to laparoscopic ovarian drilling are independent predictors of spontaneous ovulation within three months of surgery. We suggest to preferentially performing laparoscopic ovarian drilling in patients with high luteinizing hormone and androstenedione levels.

## Background

Polycystic ovary syndrome (PCOS) is the most common female endocrinopathy with an incidence of 5-10% in the female population. It is characterized by an overproduction of ovarian androgens thereby leading to symptoms such as anovulation, oligomenorrhea, hirsutism, acne, and infertility [1]. Laparoscopic ovarian drilling (LOD) is a treatment option for clomiphene citrate-resistant infertile women with PCOS. It has been reported that LOD leads to induction of spontaneous ovulation in 30-90% of women undergoing this procedure [2].

In a prospective randomized controlled trial comparing LOD to gonadotropin treatment for clomiphene citrate-resistant infertile women, Farquhar et al. reported no significant differences in pregnancy and miscarriage rates [3]. LOD is accompanied by lower direct and indirect costs and a reduction in multiple pregnancies compared to gonadotropins [4]. Therefore, LOD is a reasonable alternative to gonadotropin stimulation in clomiphene citrate-resistant infertility patients.

Up to 70% of women with PCOS respond to LOD [5]. In order to optimize the efficacy of therapeutic regimens it would be helpful to reliably predict a successful response to LOD in order to better counsel patients about this treatment option. Previous studies, published by Abdel et al. [6] and by Li et al. [7], demonstrated that patients with elevated preoperative luteinizing hormone (LH) levels had a better response to LOD compared to patients with low LH levels (> 12 IU/l and > 10 IU/l, respectively). A recent retrospective study of Hayashi et al. [8] also indicated that preoperative serum luteinizing hormone (LH) levels are a good predictor of LOD efficacy. Significantly higher preoperative LH levels were found in women who ovulated after LOD compared to those who did not ovulate. In a multiple logistic regression model, neither testosterone, nor follicle simulating hormone (FSH) or the LH/FSH ratio were associated with the likelihood of ovulation after LOD [8].

The aim of our study was to investigate LH, FSH, testosterone and androstenedione as predictive markers for therapeutic success of LOD, defined as spontaneous ovulation within three months after LOD.

## Methods

### Patient collective

In a retrospective cohort study all clomiphene citrate-resistant women with PCOS who underwent LOD at the Department of Gynecology and Obstetrics of the Medical University of Vienna, Vienna, Austria, between January 2001 and December 2008 were included. PCOS was diagnosed according to the revised European Society of Human Reproduction and Embryology (ESHRE) and American Society for Reproductive Medicine (ASRM) criteria of 2004 which were based on the Rotterdam criteria [9]. Clomiphene citrate resistance was defined as the absence of developing follicles after ovarian stimulation with 150 mg clomiphene citrate/day given for 5 days beginning with the 2^nd ^day of the menstrual cycle. Patients were stimulated with clomiphene for a minimum of three and a maximum of six cycles. A total of 121 patients who met these criteria and underwent LOD were identified, of whom 21 patients were lost to follow-up. All patients had been pretreated with metformin for at least three months. For details on characteristics of the remaining 100 patients see Table 1.

**Table 1:** Patient characteristics

Total number of patients	100
Age (years)	28.2 ± 4.7
Body mass index (kg/m^2^)	26.5 ± 4.4
Patients with primary infertility	69 (69.0%)
Patients with secondary infertility	31 (31.0%)

### Study design

The primary objective of the study was to assess independent predictors of the therapeutic success of LOD, namely spontaneous ovulation within 3 months after LOD. As a secondary objective, we evaluated the pregnancy rate within one year after LOD and the resulting life birth rate. In the course of this retrospective study, we focused on the preoperative serum levels of LH, FSH, testosterone, and androstenedione as candidates for predicting the success of LOD. These parameters are known to be altered in PCOS patients and thus might be useful for the prediction of spontaneous ovulation. Also, we included the patient's age and body mass index (BMI) in the univariate analysis, since these have previously been shown to be associated with LOD outcome [7,10]. Also, we included the type of surgical technique in the univariate analysis in order to demonstrate that this variable has no influence on the response to LOD. In addition, we evaluated the postoperative LH and androstenedione levels.

Preoperative assessment of hormonal parameters was done within three months before LOD. The clinical endpoint was the identification of at least one spontaneous ovulation within three months after LOD. Ovulation was defined as elevation of the serum progesterone level >8.0 ng/ml according to previously published studies [6,8]. Patients underwent regular follow-up examinations including ultrasound monitoring of the follicle growth and daily measurements of LH, FSH, and progesterone from the 9^th ^day of their menstrual cycle. In case a patient did not menstruate after LOD, bleeding was induced by administration of progesterone after a maximum of 40 days after LOD. Patients were followed-up after LOD with monthly visits. Patients were not allowed to use clomiphene citrate for a minimum of three months after LOD.

### Laboratory analyses

Preoperative blood samples were taken from a peripheral vein between seven days and two months before LOD. All examined serum parameters were determined in the central laboratory of the General Hospital of Vienna, Vienna, Austria using commercially available assays. Radioimmunoassays were used to determine serum levels of LH (Autodelfia; Wallac Oy, Turku, Finland), FSH (Enzymun ES700; Böhringer Mannheim, Mannheim, Germany), progesterone (Coat-ACoat RIA; DPC, Los Angeles, CA), testosterone (Immunotech, Westbrook, ME, USA), and androstenedione (Immunotech, Westbrook, ME, USA).

### Surgical technique

Ninety-one patients were treated with bilateral LOD using a monopolar electrocoagulation technique as previously reported [11]. Thirty patients were operated with a monopolar hook electrode (GK 375R, B. Braun Aesculap, Maria Enzersdorf, Austria) and 5-10 incisions of 2-3 mm length of the ovarian capsule.

The study was approved by the Ethics Committee of the Medical University of Vienna, Vienna, Austria (Internal Review Board number 388/2009). All procedures were carried out in accord with the "Good Scientific Practice Standards" set forth by the Medical University of Vienna, which are based on the ethical standards of the Helsinki Declaration of 1975.

### Statistical analysis

Variables are described by frequencies and mean ± standard deviation (SD). Since there are 71 events in our study, i.e. spontaneous ovulation after LOD, we tested 7 parameters by use of a univariate followed by a multivariate regression model in order to identify factors predictive for spontaneous ovulation after LOD. All parameters were tested as continuous variables except for surgical technique which was tested as a categorial variable.

The optimal cut-off of the investigated parameters was calculated automatically based on the receiver-operator characteristics (ROC) curve by use of the MedCalc™ (version 10.4.8.0) software as the threshold value with the highest specificity and sensitivity. The discriminatory ability of the investigated parameters is described as the correlation of specificity and sensitivity and is measured by the area under the receiver-operating (AUC) curve. All values are given with 95% confidence interval (95% CI).

Pre- and postoperative LH and androstenedione levels were compared by means of paired t-tests. A p-value < 0.05 was considered statistically significant.

## Results

One hundred of 121 patients (82.6%) underwent regular follow-up examinations as described in the methods section within three months after LOD. Twenty-one patients were lost to follow-up and were excluded from the study. Characteristics of the remaining 100 patients are shown in Table 1. Thirty-two of 100 patients became spontaneously pregnant within three months after LOD. Pregnancy was confirmed by determination of β-HCG serum levels and vaginal ultrasound.

Preoperative measurement of hormonal parameters was done 32.9 ± 9.1 days before LOD. Table 2 shows the details of preoperative hormone levels of all 100 study patients. Spontaneous ovulation within three months after LOD was found in 71/100 patients (71.0%). Patients ovulated for the first time within 37.8 ± 10.4 days after LOD. Differences in patient characteristics and hormonal parameters between patients with and without spontaneous ovulation after LOD are listed in Table 3. We used a univariate and a multivariate regression model in order to identify independent predictors of spontaneous ovulation after LOD. In the multivariate analysis, LH and androstenedione levels were significantly associated with spontaneous ovulation after LOD (p < 0.001 and p = 0.019, respectively). Details are given in Table 4.

**Table 2:** Preoperative measurement of hormonal parameters

Hormones	
Luteinizing hormone (IU/l)	15.1 ± 6.0
Follicle stimulating hormone (IU/l)	6.2 ± 1.7
Testosterone (ng/ml)	0.8 ± 0.4
Androstendione (ng/ml)	3.2 ± 0.9

**Table 3:** Patient characteristics and preoperative parameters in patients with and without spontaneous ovulation

	Spontaneous ovulation (n = 71)	Persistence of anovulation (n = 29)	*P *value
Age (years)	26.2 ± 4.4	27.1 ± 4.2	n.s.^a^
Body mass index (kg/m^2^)	27.9 ± 4.4	28.8 ± 5.4	n.s.^a^
*Surgical technique:*			
Standard monopolar electrocoagulation Monopolar hook electrode	49 (69.0%) 22 (31.0%)	21 (72.4%) 8 (27.6%)	n.s.^a^
Luteinizing hormone (IU/l)	17.3 ± 5.3	9.9 ± 4.1	<.001
Follicle stimulating hormone (IU/l)	6.4 ± 1.6	5.8 ± 1.9	n.s.^a^
Testosterone (ng/ml)	0.8 ± 0.4	0.7 ± 0.3	n.s.^a^
Androstendione (ng/ml)	3.4 ± 1.0	2.9 ± 0.7	.018

^a ^n.s. = not significant

**Table 4:** Variables associated with induction of spontaneous ovulation

	Univariate analysis			Multivariate analysis		
Variable	Odds ratio	95% CI^a^	*P *value	Odds ratio	95% CI^a^	*P *value
Age	0.96	[0.87; 1.05]	.384			
Body mass index	0.96	[0.87; 1.05]	.373			
Surgical technique	0.85	[0.33; 2.21]	.737			
Luteinizing hormone	1.51	[1.28; 1.78]	<.001	1.58	[1.30; 1.92]	<.001
Follicle stimulating hormone	1.27	[0.97; 1.66]	.078			
Testosterone	3.31	[0.73; 15.09]	.122			
Androstendione	2.18	[1.14; 4.14]	.018	3.03	[1.20; 7.67]	.019

^a^CI = confidence interval

For LH the AUC of the ROC curve was 0.88 (95% CI: 0.80 - 0.94), for androstenedione the AUC of the ROC curve was 0.65 (95% CI: 0.55 - 0.75). In order to maximize the sum of positive and negative predictive values, a cut-off of 12.1 IU/l for LH and of 3.26 ng/ml for androstenedione were evaluated. Sixty-three of 70 (90.0%) and 36/42 (85.7%) patients with elevated LH and androstenedione levels, respectively, ovulated spontaneously after LOD. Among patients with LH and androstenedione below the cut-off, spontaneous ovulation was found in 8/30 (26.7%) and 35/58 cases (60.3%), respectively. When both LH and androstenedione were analysed together, 0/31 patients (0.0%) with both LH and androstenedione exceeding the cut-off points and 16/19 patients (84.2%) with LH and androstenedione below the cut-off point remained anovulatory. Sensitivity, specificity, positive predictive value (PPV), and negative predictive value (NPV) to predict spontaneous ovulation are given in Table 5.

**Table 5:** Accuracy of LH and androstendione cut-off points to predict spontaneous ovulation and life birth

	Spontaneous ovulation (n = 100)			Life birth (n = 71)		
	LH >12.1 IU/l	Androstenedione >3.26 mg/dl	LH >12.1 IU/l and androstenedione >3.26 mg/dl	LH >12.1 IU/l	Androstenedione >3.26	LH >12.1 IU/l and androstenedione >3.26 mg/dl
Sensitivity (%)	**88.7 **(95% CI 79.0-95.0)^a^	**50.7 **(95% CI 38.6-62.8)^a^	**43.7 **(95% CI: 31.4-55.3)^a^	**88.9 **(95% CI: 73.9-96.9)	**44.4 **(95% CI: 27.9-61.9)	**36.1 **(95% CI: 20.2-52.5)
						
Specifity (%)	**75.9 **(95% CI 56.5-89.7)^a^	**79.3 **(95% CI 60.3-92.0)^a^	**100.0 **(95% CI: 85.0-100.0)^a^	**54.3 **(95% CI: 36.6-71.2)	**42.9 **(95% CI: 26.3-60.6)	**100.0 **(95% CI: 87.4-100.0)
						
Positive predictive value (%)	**90.0 **(95% CI 80.5-95.8)^a^	**85.7 **(95% CI 71.5-94.6)^a^	**100.0 **(95% CI: 85.9-100.0)^a^	**66.7 **(96% CI: 51.6-79.6)	**44.4 **(95% CI: 27.9-61.9)	**100.0 **(95% CI: 69.8-100.0)
						
Negative predictive value (%)	**73.3 **(95% CI 54.1-87.7)^a^	**39.7 **(95% CI 27.0-53.4)^a^	**42.0 **(95% CI: 29.8-53.8)^a^	**82.6 **(95% CI: 61.2-95.0)	**42.9 **(95% CI: 26.3-60.6)	**60.3 **(95% CI: 45.8-71.9)

^a^CI = confidence interval

Complete 1-year follow-up was available for 74/100 patients (74.0%). Twenty-two of 71 patients (31.0%) with spontaneous ovulation in contrast to 4/29 patients without spontaneous ovulation (13.8%) were lost to follow-up (p = 0.075). Of the 74 patients with follow-up, three women became pregnant after in-vitro fertilization within one year after LOD and thus they were excluded from the pregnancy rate and life birth rate analyses.

Fourty-one women became spontaneously pregnant and two women became pregnant after clomiphene citrate stimulation within one year after LOD resulting in a one-year pregnancy rate of 43/71 (60.6%). Of these, 36 pregnancies resulted in a life birth (36/71; 50.7%). Considering life-birth after spontaneous conception as a clinical endpoint, 32/48 (66.7%) and 16/36 (44.4%) patients with elevated LH and androstenedione levels, respectively, gave birth to a child. Among patients with LH and androstenedione below the cut-off, life birth was observed in 4/23 (17.4.7%) and 20/35 women (57.1%), respectively.

All 13 patients with both LH and androstenedione above the respective cut-offs achieved a life birth, whereas only 33/58 (39.7%) women with LH or androstenedione below the cut-offs gave birth to a child. Sensitivity, specificity, PPV, and NPV of LH and androstenedione to predict life birth are given in Table 5.

Table 6 gives an overview of pre- and postoperative LH and androstenedione levels. Postoperative LH and androstenedione levels were available in 82/100 patients (82.0%). The first postoperative measurement of hormonal parameters was done 30.1 ± 17.1 days after the operation. Statistically significant declines of both parameters were found in all patient groups, except in those with LH levels below the cut-off: LOD did not lead to a reduction of androstenedione levels in these patients.

**Table 6:** Pre- and postoperative LH and androstenedione levels

		LH			Androstenedione		
		Preoperative	Postoperative	P	Preoperative	Postoperative	P
**Total study population**		15.1 ± 6.0	6.4 ± 4.0	< 0.001	3.2 ± 0.9	2.2 ± 1.6	< 0.001
**LH**	≤12.1 IU/l	8.4 ± 2.2	6.1 ± 3.3	0.004	3.1 ± 1.0	3.0 ± 2.7	0.708
	>12.1 IU/l	18.0 ± 4.6	6.6 ± 4.3	< 0.001	3.2 ± 0.9	1.9 ± 0.7	< 0.001
**Androstenedione**	≤3.26 mg/dl	14.5 ± 5.4	6.5 ± 4.3	< 0.001	2.6 ± 0.5	1.8 ± 0.6	< 0.001
	>3.26 mg/dl	16.0 ± 6.7	6.4 ± 3.5	< 0.001	6.4 ± 0.9	2.7 ± 2.3	< 0.001
**LH and androstenedione**	LH ≤ 12.1 IU/l or androstenedione ≤ 3.26 mg/dl	13,7 ± 5,4	6,6 ± 4,0	< 0.001	2.8 ± 0.8	2.3 ± 2.0	0.029
	LH >12.1 IU/l and androstenedione >3.26 mg/dl	18,3 ± 5,9	6,1 ± 4,0	< 0.001	3.9 ± 0.8	2.0 ± 0.6	< 0.001

## Discussion

In this retrospective study of 100 women with clomiphene citrate-resistant PCOS, preoperative LH and androstenedione levels were found to be independent predictors of spontaneous ovulation after LOD.

LH is known to stimulate ovarian theca cells to produce androstenedione. Additionally, it is responsible for ovulation and luteinization. Elevated levels of LH are characteristic for women with PCOS [12]. In our study collective, significantly higher preoperative LH levels were found in responders to LOD compared to non-responders. This is in accordance with a study published by Hayashi et al. [8] demonstrating a good ovulation response in women with clomiphene citrate-resistant PCOS when the preoperative LH level was higher than 8.0 IU/l (sensitivity 73%, positive predictive value 92%). By setting the LH cut-off to 12.1 IU/l in our study, a sensitivity of 89% and a positive predictive value of 90% were reached. This cut-off is similar to that reported by Abdel et al. (12 IU/l) [6], whereas Li et al. reported that 10 IU/l was an optimal cut-off for the prediction of LOD success [7].

Our study adds androstenedione as an independent predictive parameter. PCOS patients who ovulated after LOD showed significantly higher preoperative androstenedione levels than patients who did not respond to LOD (3.4 ± 1.0 vs. 2.9 ± 0.7 ng/ml, respectively; p = 0.02). This is somewhat contradictory to a study published in 1985. Aakvard and Gjonnaess reported that women ovulating after electrocauterization of the ovarian capsule showed lower androstenedione levels prior to surgery [13]. However, these patients had not been pretreated with metformin which might explain the difference between their and our results: Metformin treatment is known to lead to a reduction of testosterone but not androstenedione in women with PCOS [14]. It has been demonstrated that metformin reduces messenger RNA expression and activity of aromatase/CYP19, an enzyme specifically metabolizing androstenedione [15].

By combining both parameters, i.e. LH and androstenedione, we were able to optimize the accuracy for predicting ovulation after LOD, reaching both a specifity and a positive predictive value of 100%.

Complete one-year follow-up was available for 74% of our patients. Notably, 31% of women with spontaneous ovulation in contrast to 14% of women, who remained anovulatory, were lost to follow-up. Although this difference failed to reach significance (p = 0.07), this finding may be considered a hint that preferentially patients in whom LOD had led to a therapeutic success have not been followed-up, a fact that might has introduced bias. Another possible source of bias is surgical technique. It is of note that in 30 patients (30%) LOD was performed by use of a monopolar hook electrode, a technique that has not been published so far. However, univariable regression demonstrated that the type of surgical technique did not influence the response to LOD (p = 0.7).

In our data set, LOD led to a one-year pregnancy rate of 61% which is comparable to the majority of publications reporting pregnancy rates of 50-60% after LOD [16]. The resulting life birth rate was 51% in our study. Due to the fact that 29% of patients were lost to follow-up, we chose the pregnancy and life birth rates as secondary objectives only. The LH and androstendione cut-off points were evaluated to predict spontaneous ovulation. Nonetheless, the LH cut-off point of 12.1 IU/l also seems to be a valuable tool for the prediction of life birth after LOD with a sensitivity of nearly 90% and a negative predictive value of about 82%, whereas we consider the use of the androstenedione cut-off of 3.26 mg/dl inaccurate, since its sensitivity, specifity, negative and positive predictive values for life birth were about 43% only. In contrast, the combination of both parameters led to a specificity and a positive predictive value of 100% to predict life birth.

LOD puts the patient at risk for ovarian damage. A reduction in the ovarian reserve and, in the worst case, ovarian failure have been reported to be rare but possible complications of surgery for PCOS [17,18]. In addition, adhesion formation with subsequent impairment of fertility may occur after LOD [18]. With these serious complications in mind, we consider preoperative LH and androstenedione levels potentially helpful tools which allow for the prediction of the therapeutic outcome of LOD. Accordingly, LH and androstendione levels might be helpful to detect those patients who will most likely benefit from LOD. In this respect, putting patients who might not benefit from LOD at risk of ovarian failure and surgical complications could be avoided.

None of the other tested parameters showed a significant association with the response to LOD. Neither age, nor BMI levels were different comparing ovulating and non-ovulating patients. In previous studies, it has been reported that LOD outcome was better in younger patients and in those with a normal body mass index [7,10], a finding which we cannot confirm. As a possible explanation, it might be argued that this difference was due to the fact that all of our patients had been pretreated with metformin.

LOD markedly decreased postoperative LH and androstenedione levels demonstrating its effectiveness. This was especially true for patients with androstenedione levels >3.26 ng/ml and for patients with both LH and androstenedione exceeding the cut-off.

Our data should only be applied to clomiphene citrate-resistant PCOS women who had been pretreated with metformin. Whether higher androstenedione levels might predict spontaneous ovulation after LOD remains open. We consider this a study limitation.

## Conclusions

We consider LH and androstenedione levels simple and valuable tools in predicting the therapeutic success of LOD in patients with clomiphene citrate-resistant PCOS pretreated with metformin. We suggest to preferentially performing LOD in patients with high LH and androstenedione levels.

## Competing interests

The authors declare that they have no competing interests.

## Authors' contributions

All authors conceived and designed the study. JO, SW and CBT supervised the data collection, assisted in the analysis and drafted the manuscript; JO and SW assisted in collection and maintenance of the data; all authors assisted in conceiving, designing and analysis, and edited the manuscript. All authors read and approved the final manuscript.
